# Psychosocial Impact of Predictive Genetic Testing in Hereditary Heart Diseases: The PREDICT Study

**DOI:** 10.3390/jcm9051365

**Published:** 2020-05-06

**Authors:** Céline Bordet, Sandrine Brice, Carole Maupain, Estelle Gandjbakhch, Bertrand Isidor, Aurélien Palmyre, Alexandre Moerman, Annick Toutain, Linda Akloul, Anne-Claire Brehin, Caroline Sawka, Caroline Rooryck, Elise Schaefer, Karine Nguyen, Delphine Dupin Deguine, Cécile Rouzier, Gipsy Billy, Krystelle Séné, Isabelle Denjoy, Bruno Leheup, Marc Planes, Jean-Michael Mazzella, Stéphanie Staraci, Mélanie Hebert, Elsa Le Boette, Claire-Cécile Michon, Marie-Lise Babonneau, Angélique Curjol, Amine Bekhechi, Rafik Mansouri, Ibticem Raji, Jean-François Pruny, Véronique Fressart, Flavie Ader, Pascale Richard, Sophie Tezenas du Montcel, Marcela Gargiulo, Philippe Charron

**Affiliations:** 1 APHP, Referral Center for hereditary heart disease, Department of Genetics, Pitié-Salpêtrière University Hospital, 75013 Paris, France; carole.maupain@aphp.fr (C.M.); estelle.gandjbakhch@aphp.fr (E.G.); stephanie.staraci@aphp.fr (S.S.); pro.mel.hebert@gmail.com (M.H.); angelique.curjol@aphp.fr (A.C.); amine.bekhechi@aphp.fr (A.B.); rafik.mansouri@inserm.fr (R.M.); ibticemraji@hotmail.com (I.R.); jean-francois.pruny@aphp.fr (J.-F.P.); 2 Sorbonne Université, INSERM, Institut Pierre Louis d’Epidémiologie et de Santé Publique, F75013 Paris, France; sandrine.brice@inserm.fr; 3 APHP, department of cardiology, Pitié-Salpêtrière University Hospital, 75013 Paris, France; 4 ACTION Study Group, Pitié-Salpêtrière University Hospital, 75013 Paris, France; 5 Sorbonne Université, INSERM, UMRS 1166 and ICAN Institute for Cardiometabolism and Nutrition, 75013 Paris, France; 6 Department of Genetics, Nantes University Hospital, 44000 Nantes, France; bertrand.isidor@chu-nantes.fr; 7 APHP, department of Genetics, Ambroise Paré University Hospital, 92100 Boulogne-Billancourt, France; aurelien.palmyre@aphp.fr; 8 Department of Genetics, Lille University Hospital, Jeanne de Flandre Hospital, 59000 Lille, France; alexandre.moerman@chru-lille.fr; 9 Department of Medical Genetics, Tours University Hospital, 37044 Tours, France; annick.toutain@univ-tours.fr; 10 Department of Medical Genetics, Rennes University Hospital, 35000 Rennes, France; linda.akloul@chu-rennes.fr; 11 Normandie University, UNIROUEN, Inserm U1245 and Rouen University Hospital, Department of Genetics and Reference Center for Developmental Disorders, Normandy Center for Genomic and Personalized Medicine, F 76000 Rouen, France; anne-claire.brehin@chu-rouen.fr; 12 Medical Genetics Unit, FHU TRANSLAD and GIMI Institute, Dijon University Hospital, 21000 Dijon, France; caroline.sawka@chu-dijon.fr; 13 Department of Medical Genetics, CHU Bordeaux, Bordeaux, France, F-33000 Bordeaux, France; caroline.rooryck-thambo@chu-bordeaux.fr; 14 Department of Genetics, Strasbourg University Hospital, Institut de Génétique Médicale d’Alsace, 67200 Strasbourg, France; elise.schaefer@chru-strasbourg.fr; 15 Department of Medical Genetics, APHM, Timone Hospital, Marseille Medical Genetics, Aix Marseille University, 13000 Marseille, France; karine.nguyen@ap-hm.fr; 16 Department of Medical Genetics, Toulouse University Hospital, 31300 Toulouse, France; dupin-deguine.d@chu-toulouse.fr; 17 Department of Medical Genetics, Université Côte d’Azur, CHU, Inserm, CNRS, IRCAN, 06000 Nice, France; rouzier.c@chu-nice.fr; 18 Department of Medical Genetics, Centre Hospitalo-Universitaire Grenoble Alpes, 38700 Grenoble, France; gbilly@chu-grenoble.fr; 19 Clinical Genetics Unit, University Hospital, Guadeloupe University Hospital, 97159 Guadalupe Island, France; krystelle.sene@chu-guadeloupe.fr; 20 APHP, Department of cardiology, Referral Center for hereditary heart disease, Bichat Hospital, 75018 Paris, France; isabelle.denjoy@aphp.fr; 21 Department of Medical Genetics, University Hospital, 54042 Nancy, France; b.leheup@chu-nancy.fr; 22 Department of Medical Genetics, University Hospital Morvan, 29200 Brest, France; marc.planes@chu-brest.fr; 23 APHP, Department of Medical Genetics, Hôpital Européen Georges Pompidou, 75015 Paris, France; jean-michael.mazzella@aphp.fr; 24 Department of Genetics, Saint Brieuc Hospital, 22000 Saint-Brieuc, France; elsa.leboette@ch-stbrieuc.fr; 25 Filière nationale de santé CARDIOGEN, Pitié-Salpêtrière University Hospital, 75013 Paris, France; clairececile.michon@gmail.com (C.-C.M.); psy.cardiogen.psl@aphp.fr (M.-L.B.); 26 APHP, UF Molecular Cardiogenetics and Myogenetics, Pitié-Salpêtrière University Hospital, 75013 Paris, France; vero.fressart@aphp.fr (V.F.); flavie.ader@aphp.fr (F.A.); pascale.richard@aphp.fr (P.R.); 27 Faculté de Pharmacie Paris Descartes, Département 3, 75006 Paris, France; 28 Sorbonne Université, INSERM, Institut Pierre Louis d’Epidémiologie et de Santé Publique, AP-HP, Hôpitaux Universitaires Pitié-Salpêtrière—Charles Foix, F75013 Paris, France; sophie.tezenas@aphp.fr (S.T.d.M.); marcela.gargiulo@parisdescartes.fr (M.G.); 29 Institut of Myologie, Pitié-Salpêtrière University Hospital, 75013 Paris, France

**Keywords:** hereditary heart diseases, predictive genetic testing, psychological, social, cardiomyopathies, anxiety, distress

## Abstract

Predictive genetic testing (PGT) is offered to asymptomatic relatives at risk of hereditary heart disease, but the impact of result disclosure has been little studied. We evaluated the psychosocial impacts of PGT in hereditary heart disease, using self-report questionnaires (including the State-Trait Anxiety Inventory) in 517 adults, administered three times to the prospective cohort (PCo: *n* = 264) and once to the retrospective cohort (RCo: *n* = 253). The main motivations for undergoing PGT were “to remove doubt” and “for their children”. The level of anxiety increased between pre-test and result appointments (*p* <0.0001), returned to baseline after the result (PCo), and was moderately elevated at 4.4 years (RCo). Subjects with a history of depression or with high baseline anxiety were more likely to develop anxiety after PGT result (*p* = 0.004 and *p* <0.0001, respectively), whatever it was. Unfavourable changes in professional and/or family life were observed in 12.4% (PCo) and 18.7% (RCo) of subjects. Few regrets about PGT were expressed (0.8% RCo, 2.3% PCo). Medical benefit was not the main motivation, which emphasises the role of pre/post-test counselling. When PGT was performed by expert teams, the negative impact was modest, but careful management is required in specific categories of subjects, whatever the genetic test result.

## 1. Introduction

Advances in molecular genetics in numerous hereditary diseases have enabled the development of pre-symptomatic or predictive genetic testing (PGT), which raises various medical challenges depending on the disease and the treatment resources [1]. PGT raises complex issues, not only medical but also psychological, ethical, and sometimes social or professional [2,3]. In neurodegenerative diseases, which generally do not involve therapeutic issues, several studies [4,5,6,7,8] have shown that disclosure of genetic test results may lead to serious adverse events (depression, suicide or attempted suicide) which show that great care should be exercised in PGT, through psychosocial support and counselling and codified procedures [9].

Hereditary heart diseases [10,11] fall into two main groups: (i) cardiomyopathies such as hypertrophic cardiomyopathy, dilated cardiomyopathy, restrictive cardiomyopathy, and arrhythmogenic right ventricular cardiomyopathy; and (ii) isolated arrhythmias, such as long QT syndrome and Brugada syndrome. These diseases have a prevalence of 1/2500 to 1/5000 for the main ones, and 1/500 for the most frequent (hypertrophic cardiomyopathy). They are associated with a risk of heart failure and/or arrhythmia which may result in sudden death. The mode of transmission is usually autosomal dominant, and these diseases are characterised by age-related increase in penetrance, with phenotypic expression often delayed until adulthood. Molecular understanding in this field [10,11] has led to increasing development of clinical genetic testing and international guidelines recommending PGT of asymptomatic relatives at risk of hereditary heart disease [12,13,14]. PGT identifies those who do not carry the familial mutation, who can therefore be reassured and for whom cardiovascular monitoring can be stopped. Equally, PGT identifies those who do carry the mutation and who therefore need targeted medical management via regular cardiovascular follow-up to ensure prompt and optimal treatment.

However, very few studies have investigated the medical and psychosocial impact of PGT in hereditary heart disease [15,16,17,18,19]. Our aim was therefore to assess the psychological, social or professional impact of disclosure of genetic status specifically in asymptomatic relatives at risk of hereditary heart disease who have undergone PGT.

We hypothesised that: (i) the main reason for PGT is the medical benefit provided by knowledge of genetic status, (ii) the psychological, social or professional impact can be harmful and may worsen over time, (iii) an unfavourable impact is linked to a family history of severe disease, competitive sporting activities, a high-risk profession, and/or a mismatch between the subjective risk and the test result.

## 2. Methods

### 2.1. Population

This French, prospective, longitudinal, multicentre study assessing the short-term (prospectively) and long-term (retrospectively) impact of PGT was approved by an ethics committee and by the French regulatory authorities (CPP, CCTIRS, CNIL). Inclusion in the study was offered to adult (18 years and above) members of families with inherited heart diseases and identified pathogenic variants in the index case who wished to undergo PGT for the cardiac disease concerned (prospective cohort or PCo) or who had in the past undergone PGT (retrospective cohort or RCo). All subjects agreed to participate in the study. The study had no impact on the modalities of genetic testing, which was done in the framework of standard care and according to the procedure specific to each of the 20 multidisciplinary (possibility of seeing a psychologist, genetic counsellor, cardiologist) expert centres (all clinical genetics departments except for one cardiology department) belonging to “Filière CARDIOGEN”, the national network for inherited or rare heart diseases which is approved by the French Ministry of Health. Only pathogenic and probably pathogenic variants in genes described in inherited heart diseases were considered for PGT, according to local management in daily practice. Before the study began, the various centres took part in a study set-up meeting.

### 2.2. Questionnaires

The study subjects’ opinions were collected using paper self-report questionnaires comprising validated anxiety and distress scores as well as 24 home-made items in the PCo and 35 home-made items in the RCo. These home-made items related to: reasons for PGT, perception of heart disease, perception of genetic risk, family context, degree of anxiety, social or professional impact (change in professional activity, change in sporting activity, change in family planning, negative impact on bank loan application, change in family relationships).

The State-Trait Anxiety Inventory (STAI) [20] was used to evaluate anxiety. It is divided into two parts: one for “state anxiety” (i.e., emotional reaction at the time of the survey) and the other for “trait anxiety” (i.e., level of anxiety during daily life). Each part has twenty questions scored from 1 to 4. Anxiety is considered significant if the global score is above 35, high if it is above 55, and very high above 65. The French version was validated by Bruchon-Schweitzer and Paulhan [21]. We used the STAI trait score to compare the level of anxiety during daily life of our cohort to the anxiety of the general population in France. With the STAI state score we measured anxiety before and after PGT.

The Impact of Event Scale (IES, total score) [22] is a set of 15 questions that measure the amount of distress associated with a specific event. The IES is often useful in measuring the impact of the experience following a traumatic event. It is valuable in spotting both trauma and less intense forms of stress and will show how much an impact event is currently bothering the subject. The IES is also used to detect the effect of the most severe impact events: 0–8: no meaningful impact, 9–25: mild impact, 26–43: moderate impact, 44–75: severe impact.

In the prospective study, subjects included between 19 February 2016 and 18 February 2017 completed three self-report questionnaires: the first (Qp1) was completed in the department just before the first appointment, the second (Qp2) was completed in the department just before disclosure of the test results, and the third and last (Qp3) was sent to the study subject’s home address two to three months after he/she received the test results (Qp3 was then completed and subsequently send by post to investigators). We excluded subjects who did not complete the procedure (sampling not done or failure to collect the test results) and who did not return the questionnaire Qp3.

In the retrospective study, the subjects had undergone PGT between January 2000 and February 2016 and were included between 19 February 2017 and 18 April 2017. If accepted, a paper self-report questionnaire was sent to their home address (Qr).

### 2.3. Statistical Analysis

The questionnaire data were entered in the EpiData 3.1 database (The Epidata Association, version: 3.1, Odense, Denmark), with independent double data entry to minimize the risk of input errors. Data were first summarized with frequencies and percentages for qualitative data and means and standard deviations for quantitative variables.

Univariate and multivariate analyses were performed to identify predictors of anxiety, distress and the impact of test results disclosure. Parameters studied for univariate analyses are detailed in Supplementary Table S1. To assess the association between a dependent variable and explicative variables, a logistic regression model or a linear regression model was used depending on whether the dependent variable was qualitative or quantitative. Statistical tests were performed using the conventional two-tailed type I error of 0.20 in the univariate setting and 0.05 in the multivariate setting. Data were analyzed using SAS version 9.4 (SAS Institute, Cary, NC, USA).

## 3. Results

**Figure 1:**
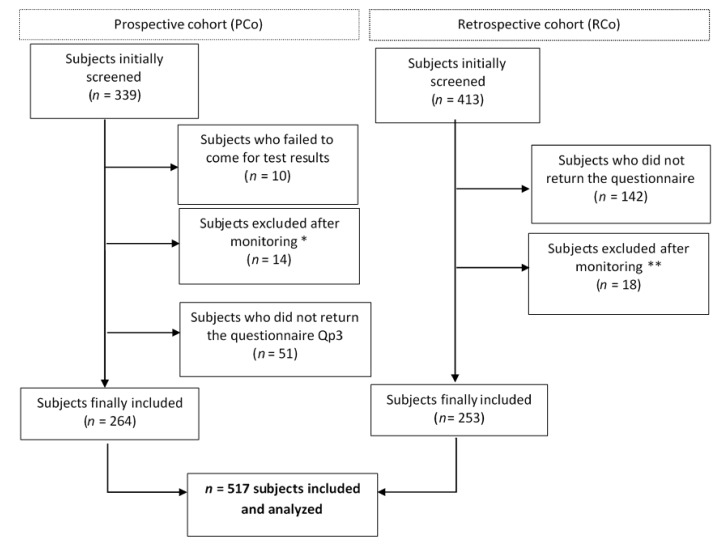
Flow chart of enrolment. * Inclusion or exclusion criteria not respected: risk of vascular disease (Marfan syndrome) (*n* = 6), abnormal cardiovascular findings (*n* = 1), no sampling after provision of information (*n* = 2), unwilling to complete questionnaire (*n* = 2), predictive genetic testing not done because mutation reclassified as a polymorphism (*n* = 3); ** Inclusion or exclusion criteria not respected: abnormal cardiovascular findings (*n* = 3), received test result by post, with no physical meeting (*n* = 6), failure to collect test result (*n* = 1), already included in the PCo (*n* = 1), interval between disclosure of test result and sending of completed questionnaire <30 days (*n* = 7).

Of 752 subjects initially screened, 517 were finally included (retrospective study *n* = 253, prospective study *n* = 264) (see flow chart in Figure 1). The questionnaires of the retrospective study were completed on average 4.4 ± 3.5 years after disclosure of the test result. In the prospective study, the questionnaire Qp3 was completed on average 5.4 ± 8.8 months after disclosure of the test result.

The average age of the subjects was 42.3 ± 16.7 years in the prospective cohort (PCo) and 43.0 ± 15.2 years in the retrospective cohort (RCo). Distribution of age of subjects according to sex is detailed in Supplementary Figure S1. The subjects were predominantly female (60.6% in PCo and 60.9% in RCo), and most subjects were at risk of cardiomyopathy (88.4% in PCo and 79.1% in RCo). After PGT, 39.4% of subjects in the prospective cohort and 49.4% of those in the retrospective cohort were found to carry the familial mutation (see Table 1 for sociodemographic characteristics).

### 3.1. Expectations From the First Provision of Information

Subjects in the prospective cohort (*n* = 264) were asked for their overall expectations regarding the pre-test appointment: 93.1% came with the intention of undergoing PGT (*n* = 243), 58.1% came for information on the disease (*n* = 151), 42.3% were responding to a request from a close relative (*n* = 110) and 4.2% were seeking support from a team (*n* = 11).

### 3.2. Reasons for Undergoing Predictive Genetic Testing

Of the subjects in the prospective cohort, 65.3% (171/262) wanted to undergo PGT to remove doubt, 64.0% (167/261) for their children (to know if they are at risk), 34.9% (91/261) to know whether they should be medically monitored, 24.4% (64/262) to prepare for the future, 23.8% (62/261) because of a close relative’s worries, and 5.3% (14/262) because of a planned pregnancy. The results for the subjects of the retrospective cohort were very similar (see Table 2).

### 3.3. Global Life Changes After Predictive Genetic Testing

Among the subjects in the prospective cohort, 18.6% (48/258) reported that PGT changed their lives, including 10 who were carriers of the mutation and 38 who were not. Among the subjects of the retrospective cohort, 23.5% (59/251) reported that PGT changed their lives, including 25 who were carriers of the mutation and 34 who were not. Details of the adjustments made by subjects whose lives were changed by PGT are shown in Supplementary Table S2. In the retrospective and prospective cohorts, PGT mainly removed subjects’ doubts (RCo: 71.2% and PCo: 89.6%) and determined the risk for their children (RCo: 61.0% and PCo: 50.0%).

### 3.4. Changes in Social, Professional or Family Relationships After Predictive Genetic Testing

**Figure 2:**
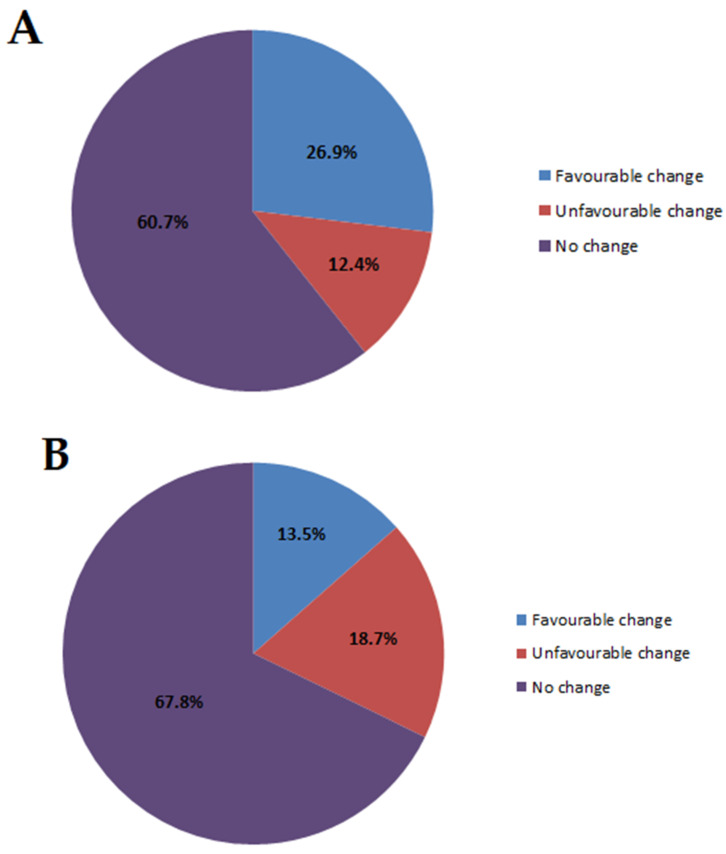
Social or professional changes and/or changes in family relationships in the prospective cohort (**A**) and retrospective cohort (**B**).

In the prospective cohort, 39.3% (92/234) of the subjects reported changes in social or professional status and/or in family relationships after PGT. Multivariate analysis showed that this change was associated with anxiety at Qp1 (baseline) (*p* = 0.011) and was associated with baseline subjective risk of being mutation carrier (*p* = 0.049). The change was considered favourable by 26.9% (63/234) of the subjects and unfavourable by 12.4% (29/234) (Figure 2A). Among those who reported an unfavourable change, 44.8% (13/29) were carriers of the familial mutation and 55.2% (16/29) were not (see Supplementary Figure S2 for detailed analyses according to genetic testing results).

In the retrospective cohort, 32.1% (81/252) of subjects experienced changes in their social or professional status or in their family relationships following PGT. Multivariate analysis showed that this change was associated with the genetic test result (*p* <0.0001). The change was considered favourable by 13.5% (34/252) of subjects and unfavourable by 18.7% (47/252) (Figure 2B). Among those who reported a mostly unfavourable change, 36/47 (76.6%) were carriers of the familial mutation and 11/47 (23.4%) were not.

Supplementary Table S3 details the changes in social or professional status and in family relationships among subjects who reported a change after PGT. In the retrospective cohort, and hence longer term, among subjects who reported changes, a change in sporting activities was observed in 65.4%, a change in the relationship of the couple in 47.1%, a change in professional plans in 14.8%, and 13.2% reported that the test result complicated an application for a bank loan.

### 3.5. Psychological Impact of Disclosure of Genetic Status

In the prospective cohort:

We found that there was a significant correlation (coefficient of correlation = 0.54, *p* <0.0001) between the STAI trait score and the STAI state score at Qp1. Subjects who were anxious at the time of PGT (STAI state score) were mostly subjects anxious during their daily lives (STAI trait score). Hereafter, therefore, we shall only detail the results concerning the STAI state score.

The level of anxiety determined using the STAI state score (see Table 3 and Supplementary Figure S3) was on average 30.5 ± 9.6 before the pre-test appointment (Qp1), 34.7 ± 12.1 before the appointment when the genetic test result was disclosed (Qp2) and 30.0 ± 10.4 sometime after this disclosure (Qp3). The difference in STAI score was significant between Qp1 and Qp2 (*p* < 0.0001) and between Qp2 and Qp3 (*p* <0.0001), but not significant between Qp1 and Qp3 (*p* = 0.616). After the genetic test result at Qp3, 23.3% (*n* = 58) of patients were anxious (STAI score >35). The factors associated with anxiety at Qp3 were studied by multivariate analysis (*n* = 197): subjects anxious at Qp1 were more at risk of being anxious at Qp3 (STAI > 35: OR 5.79, [95% CI 2.79–12.05], *p* < 0.0001), independently of the genetic test result (test interaction, *p* = 0.128). Apart from this variable, male subjects (OR 0.38, [95% CI 0.17–0.86], *p* = 0.020) and subjects practicing sports (OR 0.49, [95% CI 0.24–0.98], *p* = 0.044) were less at risk of being anxious at Qp3 (*n* = 215). Genetic status was not significantly associated with the anxiety score at Qp3 and score was 31.7 ± 11.0 in subjects carrying the mutation versus 28.9 ± 9.9 in subjects without the mutation (*p* = 0.070 by univariate logistic regression).

The level of distress assessed by the IES score (see Table 4 and Supplementary Figure S4) was on average 6.9 ± 9.8 before the pre-test appointment (Qp1), 8.7 ± 10.5 before the appointment for disclosure of the test result (Qp2) and 6.5 ± 10.0 sometime after disclosure of the result (Qp3) (*p* = 0.012 between Qp1 and Qp2, *p* <0.0001 between Qp2 and Qp3, and *p* = 0.412 between Qp1 and Qp3). Multivariate analysis (*n* = 181) showed that in subjects for whom there was no mismatch between their subjective risk (a priori) and the genetic test result, the increase in IES score between Qp1 and Qp3 was significantly less (*p* = 0.004) than in subjects for whom there was a mismatch (contradicting their a priori belief). Genetic status was not associated with a significant increase in IES score, and mean IES score at Qp3 was 7.6 ± 10.4 in subjects carrying the mutation versus 5.8 ± 9.7 in subjects without the mutation (*p* = 0.361 by univariate linear regression). These scores indicate that the genetic test result did not have a significant traumatic impact (<8) or that the impact was mild (>9).

In the retrospective cohort:

The mean STAI state score in subjects of the retrospective cohort was 35.2 ± 11.7 (Table 3). Multivariate analysis showed that the independent variables significantly associated with anxiety at Qr were a history of depression or antidepressant treatment (OR 3.27, [95% CI 1.47–7.25], *p* = 0.004) and age at the initial appointment (age ≥26 years: OR 3.01, [95% CI 1.17–7.74], *p* = 0.022), but not the genetic test result. Mean STAI score was 35.7 ± 11.7 in subjects carrying the mutation versus 34.8 ± 11.8 in subjects without the mutation (*p* = 0.256 by univariate logistic regression).

The mean IES score was 12.9 ± 14.0 (mild impact in the range >9 and <25) (Table 4). Multivariate analysis (n = 199) showed that the IES score was significantly higher in subjects with a history of depression or antidepressant treatment (*p* = 0.022), in subjects from a family with inherited heart disease other than hypertrophic cardiomyopathy (*p* = 0.015) (see Supplementary Table S4 for descriptive analysis of STAI State and IES in HCM patients versus other diseases), and in subjects carrying the genetic mutation (*p* = 0.007). Mean IES score was 15.6 ± 15.0 in subjects carrying the mutation versus 10.0 ± 12.4 in subjects without the mutation (*p* = 0.003 by univariate linear regression) (Table 4).

Apart from logistic or linear regression analyses between genetic status and anxiety or distress, additional direct comparisons were performed in the prospective and retrospective cohorts between mutation carriers and non-carriers (see Supplementary Table S5). Same results were observed without significant differences in STAI or IES mean scores related to genetic status (except IES mean score in RCo in agreement with above results, and for STAI mean score at Qp3). When STAI index >35 was considered, no significant differences were observed between mutations carriers versus non-carriers, including in RCo (44.1% versus 36.8% with anxiety respectively, *p* = 0.256 by Chi-Square test).

### 3.6. Regret at Having Undergone Predictive Genetic Testing

Regret at having undergone PGT was reported by 2.3% (6/262) of subjects in the prospective cohort and by 0.8% (1/131) of subjects in the retrospective cohort.

## 4. Discussion

To our knowledge this is the first psychosocial study after PGT in hereditary heart diseases, that simultaneously assessed the reasons for undergoing PGT, anxiety, distress and the social impact of the disclosure of genetic test results. This study was conducted in the largest population analyzed so far in cardiomyopathies and primary arrhythmic disorders (see Supplementary Table S6) [17,23,24,25,26,27] and is the only study to focus specifically on asymptomatic relatives without phenotypic expression (other studies have comprised a mixed population including a majority of patients with already manifest cardiac disease). It is one of the few longitudinal studies combining pre-test and post-test evaluation and is one of the few that examine predictors of psychosocial impact (only studied by Christiaans et al.) [24].

Contrary to what we hypothesized, the primary reason for undergoing PGT was not medical benefit or guidance of cardiological follow-up. Rather, the main reasons for testing were nonmedical and above all removal of doubt and knowing whether offspring (children or future children) are at risk. Similar results have been found with neurodegenerative disorders like Huntington’s disease [28], which is to be expected given that in such diseases the results of genetic testing will not impact on medical and therapeutic management. In hereditary heart diseases, two qualitative studies by interview in small study populations suggest, however, similar reasons (participants often underwent the test to remove uncertainty with the aim of ruling out risk to self and children) [16,29], which we confirm quantitatively in our larger study population. This shows counterintuitively that subjects’ reasons do not tally with the a priori ideas of health professionals. These results suggest that the nonmedical dimensions of PGT should be anticipated and emphasize the role of pre/post-test counselling by expert teams. The results also raise the question of the potential consequences of subjects’ adherence to recommendations regarding subsequent cardiological follow-up. It would be interesting to study the cardiological follow-up of subjects carrying the familial mutation in our cohort.

The impact of PGT in terms of global change, social or professional change and change in family relationships has been little studied in hereditary heart disease. Our results show that disclosure of genetic status leads to a decisive global change (favourable or unfavourable) in one-fifth to one-fourth of subjects (PGT “changed their life”) and that a social or professional change and/or a change in relationships within the family was observed in one-third of subjects (39.3% in the short term in PCo and 32.1% in the long term in RCo). In multivariate analysis, these changes were significantly associated with higher baseline anxiety, and baseline subjective risk to be mutation carrier, in the subjects of the prospective cohort and were associated with the genetic test result in the subjects of the retrospective cohort. Interestingly, unfavourable changes in professional and/or family life were observed in a relatively low but meaningful proportion of our population (12.4% in PCo and 18.7% RCo) and were related to sporting activity, relationships within the family, professional career, and insurance. These results are a further indication of the need for a dedicated multidisciplinary team to anticipate these nonmedical issues.

The psychological consequences of PGT were studied in our cohort using validated scales exploring anxiety and distress: the STAI (state and trait) and IES, respectively. In the prospective longitudinal cohort, we observed, as expected, that both anxiety and distress significantly increased between the pre-test appointment and results disclosure but returned to baseline in the short term after the genetic test result, without significant differences between baseline and post-test levels. Overall, levels of anxiety and psychological distress were low in our population, which is in agreement with the literature data. A systematic review among cardiovascular, neurodegenerative and cancer diseases [15] observed no significant increase in distress and anxiety, or adverse impacts on quality of life, except in Huntington’s disease, which is characterized by depressive symptoms, suicidal ideation, and hopelessness in gene carriers. Most published studies in hereditary heart diseases have related to a mixed population of mutation carriers with or without phenotypic expression, in contrast to our population. In our prospective study, we observed that a significant proportion of subjects experienced anxiety in the short term after results disclosure (23.3% had an STAI state score >35, which is considered as the threshold for meaningful anxiety), but this percentage does not differ from that found in the French general population where 21.6% of people experience this level of anxiety at any given moment during their life between 18 and 65 years of age [30]. However, we observed in the long term (mean 4.4 years after disclosure of genetic test results) in our retrospective cohort that a larger proportion of subjects had abnormal anxiety (40.4% had an STAI state score >35). There are several possible explanations for these results in the retrospective cohort (members of which differed from those of the prospective cohort): more subjects were carriers of the mutation than in the prospective cohort (49.4% versus 39.4%); the subjects were contacted a long time after genetic test result and this may have generated stress; the impact of the test result increases with time. The latter hypothesis is at odds with several studies on the long-term impact of PGT for neurodegenerative diseases or cancer predisposition showing that levels of depression and anxiety decline over time [4,6,31]. This decrease in anxiety with time has also been observed in studies in families with long QT syndrome [17]. Other follow-up studies are needed to clarify and understand anxiety and psychological distress long-term in hereditary heart diseases.

We have analyzed the predictors of the level of anxiety or distress after PGT, with a view to identifying those subjects at high risk of an unfavourable impact. In the only study to analyze these predictors after genetic testing in hereditary heart diseases [24], anxiety was associated with strong emotional reactions and the female gender. Quality of life in this mixed population of subjects with or without the cardiac phenotype was associated with the presence of symptoms and with the conviction that the presence of the mutation entails serious consequences. Our multivariate analysis shows that the level of anxiety after disclosure of the genetic test result is significantly associated with the subject’s initial level of anxiety (in the short-term prospective study) and with a history of depressive syndrome (in the long-term retrospective study). Our results also show that psychological distress after PGT is significantly associated with the mismatch between the subject’s initial subjective perception of risk and his/her genetic test result (in the prospective study). Surprisingly, our results show that anxiety after the genetic test result is not significantly associated with the result itself (carrier or not of the mutation), which suggests that subjects who do not carry the mutation are also exposed to an adverse impact and this possibility should be anticipated in patient management. Apart from cardiology field, initial anxiety was identified as a risk factor for distress after BRCA1/2 genetic testing [32]. A history of depression has also been identified as the main risk factor for post-test depression, regardless of the genetic test result, in subjects at risk for Huntington’s disease [33]. Furthermore, and contrary to our hypotheses, our results show that the level of anxiety after PGT is not associated with a family history of severe disease, the practice of competitive sports or high-risk professions.

Lastly, we found a very low level of regret among the study participants at having undergone PGT (0.8% in RCo, 2.3% in PCo), which suggests that genetic testing as practiced by experts in genetic counselling was satisfactory. This level of regret was even lower than that reported by Wynn et al. [23] in a rare cardiology study in which 79% of the subjects were completely satisfied with their decision to undergo PGT.

Taken together, these results point to various conclusions regarding the procedure and patient support associated with PGT in hereditary heart diseases. The global psychological impact evaluated in terms of anxiety and distress seems low and reassuring, but the social and familial impact is important for one-third of subjects and almost one-fifth of subjects rated the impact as unfavourable. This confirms the importance of structured support for subjects undergoing PGT provided by an expert multidisciplinary team [1,12,15,34]. Some categories of subjects at greater risk of unfavourable psychosocial impact were identified: those with a history of depression or a high baseline level of anxiety, and those whose subjective perception of risk before the disclosure is at odds with the genetic test result. These characteristics should be sought during genetic counselling and affected subjects should be provided with greater assistance and personalized psychological support. Conversely, an unfavourable psychosocial impact does not seem to be associated to the results of genetic testing, which suggests that special attention should also be paid to subjects without the familial mutation, who may experience difficulties after a negative genetic test result. Similar results have been found in neurodegenerative diseases [33] and the explanations advanced include a feeling of guilt at having escaped the disease while other relatives are ill or carriers of the mutation, and regret regarding past life decisions, notably when they reduced the scope of action through fear of the disease or of its transmission to offspring.

## 5. Limitations and Perspectives

We focused our study on a population of adults only and our results cannot be applied to minors. The issues associated with PGT in minors are more complex and the psychosocial impact may be different. It would be useful to conduct a similar study focusing specifically on minors (children and adolescents).

Most members of our cohorts were at risk of cardiomyopathies (the most prevalent diseases), and only a minority were at risk of isolated arrhythmias. The stakes associated with these two disease categories are perhaps different and it may be useful to analyze a larger cohort of families with isolated arrhythmias.

We evaluated the long-term psychosocial impact in a retrospective study, but this cannot be extrapolated as being representative of changes in short term data in the prospective cohort, because the two cohorts are independent and have different subjects. We cannot therefore affirm that the level of anxiety increases over time. A specific study of the same subjects over the short and long term would be valuable. In addition, our study did not analyze cardiological follow-up or adherence to cardiological monitoring.

## 6. Conclusions

Our results show that contrary to widespread opinion, medical benefit is not what most motivates people to seek PGT. In most cases, the adverse psychological and/or social impact was marginal or modest when PGT was performed by an expert team. However, careful management is required to identify and manage subjects at risk for increased psychological or social changes, especially those with history of depression or with a high baseline level of anxiety, whatever the genetic test result.

## Figures and Tables

**Table 1:** Sociodemographic characteristics of the cohorts.

	Prospective Cohort (*n* = 264)	Retrospective Cohort (*n* = 253)
Age	42.3 ± 16.7 years	43.0 ± 15.2 years
Gender:		
Female	160 (60.6%)	154 (60.9%)
Male	104 (39.4%)	99 (39.1%)
Familial disease:		
Hypertrophic cardiomyopathy	139 (52.9%)	119 (47.0%)
Dilated cardiomyopathy	39 (14.8%)	36 (14.2%)
Long QT syndrome	28 (10.6%)	48 (19.0%)
Brugada syndrome	0 (0.0%)	4 (1.6%)
ARVC	42 (16.0%)	40 (15.8%)
Other	15 (5.7%)	6 (2.4%)
Genetic test result:		
Carrier of the variant	104 (39.4%)	125 (49.4%)
Non-carrier	160 (60.6%)	128 (50.6%)
Sporting activity:		
Yes	132 (54.8%)	144 (56.9%)
No	109 (45.2%)	109 (43.1%)
Marital status:		
Single	65 (28.5%)	47 (24.0%)
In relationship	163 (71.5%)	149 (76.0%)

ARVC: arrhythmogenic right ventricular cardiomyopathy.

**Table 2:** Reasons for undergoing predictive genetic testing. (Multiple answers were allowed)

	Prospective Cohort *n* = 264	Retrospective Cohort *n* = 253
For children (to know if they are at risk)	167 (64.0%)	143 (56.5%)
To remove doubt	171 (65.3%)	129 (51.0%)
To benefit from medical monitoring	91 (34.9%)	93 (36.8%)
To prepare for the future	64 (24.4%)	39 (15.4%)
At the request of a close relative	62 (23.8%)	59 (23.3%)
Because of a planned pregnancy	14 (5.3%)	29 (11.5%)
To participate in a research protocol	49 (18.8%)	43 (17.0%)
I don’t know	5 (1.9%)	2 (0.8%)
Other	48 (18.4%)	19 (7.5%)

**Table 3:** State-Trait Anxiety Inventory state score over time.

State-Trait Anxiety Inventory state score						
	All Subjects *		Non-Carriers		Mutation Carriers	
	Mean ± SD	>35 (*n*)	Mean ± SD	>35 ^&^ (*n*)	Mean ± SD	>35 ^&^ (*n*)
Qp1	30.5 ± 9.6	68 (28.6%)	30.5 ± 9.3	41 (28.5%)	30.6 ± 10.1	27 (28.7%)
Qp2	34.7 ± 12.1	90 (39.5%)	34.9 ± 12.7	55 (40.1%)	34.4 ± 11.2	35 (38.5%)
Qp3	30.0 ± 10.4	58 (23.3%)	28.9 ± 9.9	29 (19.3%)	31.7 ± 11.0	29 (29.3%)
Qr	35.2 ± 11.7	92 (40.4%)	34.8 ± 11.8	43 (36.8%)	35.7 ± 11.7	49 (44.1%)

* Qp1 vs. Qp2: *p*-value <0.0001; Qp2 vs. Qp3: *p*-value < 0.0001; Qp1 vs. Qp3: *p*-value = 0.616. SD = standard deviation. ^&^ no association between genetic status and STAI (mean or >35) at a given time, including for Qr, except for mean score at Qp3 (*p* = 0.036 by Student’s *t*-test for comparison between mutation carriers and non-carriers).

**Table 4:** Impact of Event Scale score over time.

	Impact of Event Scale Score		
	All Subjects *	Non-Carriers ^&^	Mutation Carriers ^&^
	Mean ± SD	Mean ± SD	Mean ± SD
Qp1	6.9 ± 9.8	6.8 ± 10.7	7.0 ± 8.1
Qp2	8.7 ± 10.5	9.1 ± 11.1	8.0 ± 9.5
Qp3	6.5 ± 10.0	5.8 ± 9.7	7.6 ± 10.4
Qr	12.9 ± 14.0	10.0 ± 12.4	15.6 ± 15.0

* Qp1 vs. Qp2: *p*-value = 0.012; Qp2 vs. Qp3: *p*-value < 0.0001; Qp1 vs. Qp3: *p*-value = 0.412. SD = standard deviation. ^&^ no association between genetic status and IES in PCo (as a whole or by direct comparison at a given time) but significant association in RCo (*p* = 0.007 by multivariate linear regression and *p* = 0.003 by Student’s *t*-test).

## Supplementary Materials

The following are available online at https://www.mdpi.com/2077-0383/9/5/1365/s1, Table S1: Parameters used in univariate analysis to determine predictors of anxiety, Impact of Event Scale scores, and impact of genetic test results; Table S2: Global change after predictive genetic testing: details of changes for subjects who reported that the genetic test changed their lives; Table S3: Details of the changes in social or professional status and in family relationships for subjects who experienced change; Table S4: Descriptive analysis of STAI and distress (IES) in HCM patients versus other diseases; Table S5: Direct comparisons between mutation carriers and non-carriers; Table S6: Summary of main studies of the psychosocial impact of predictive genetic testing in hereditary heart diseases (restricted to cardiomyopathies and arrhythmias). PGT: predictive genetic testing; Figure S1: Distribution of age of subjects according to sex in the prospective cohort (A) and retrospective cohort (B); Figure S2: Social or professional changes and/or changes in family relationships in the prospective cohort (A) and retrospective cohort (B) for mutation carriers and non-carriers; Figure S3: Change in the State-Trait Anxiety Inventory state score at Qp1, Qp2 and Qp3, and the STAI state score at Qr and Figure S4: Change in the Impact of Event Scale score at Qp1, Qp2 and Qp3, and the IES score at Qr.
