# Supplementary material for: Psychosocial Impact of Predictive Genetic Testing in Hereditary Heart Diseases: The PREDICT Study
Source: J Clin Med. 2020 May 6;9(5):1365. doi: 10.3390/jcm9051365 (PMC7290753; doi:10.3390/jcm9051365)
Supplement: Supplementary file 1 [file jcm-09-01365-s001.pdf]

**SUPPLEMENTARY DATA:**

**Table S1.** Parameters * used in univariate analysis to determine predictors of anxiety, Impact of Event Scale scores, and impact of genetic test results.

| |
|---|
| Age at first appointment |
| Sex |
| History of depression or antidepressant treatment (yes versus no) |
| Seriousness of the familial disease (major cardiac clinical event in the family [&]) |
| Subjective representation of risk |
| State-Trait Anxiety Inventory state score at QP1 |
| Impact of Event Scale score at QP1 |
| Familial disease (HCM *versus* other; and cardiomyopathy *versus* other) |
| Profession deemed high-risk for someone with the disease expressed |
| Practice of sports (yes versus no) |
| Family situation (single versus couple) |
| Was accompanied at least once in request for predictive genetic testing |
| Family informed (or not) of the request for predictive genetic testing |
| Genetic test result (presence or absence of the variant) |
| Mismatch between the subjective risk and the genetic test result |
| Development of cardiac symptoms |

* Additional parameters related to the structure of the medical teams and consultation process will be analysed separately in a dedicated work

[&] Cardiac events that were considered: SCD < 50 years, heart failure death < 50 years or Heart transplantation.

**Table S2.** Global change after predictive genetic testing: details of changes for subjects who reported that the genetic test changed their lives.

| Variable | Prospective (*n* = 48) Frequency (%) | Retrospective (*n* = 59) Frequency (%) |
|---|---|---|
| **The test result enabled you to** | | |
| Prepare for the future | 13 (27.1%) | 12 (20.3%) |
| Benefit from suitable medical monitoring | 9 (18.8%) | 27 (45.8%) |
| Remove doubt | 43 (89.6%) | 42 (71.2%) |
| Take part in research | 14 (29.2%) | 13 (22.0%) |
| Know whether your children are at risk | 24 (50.0%) | 36 (61.0%) |
| Guide your family planning | 7 (14.6%) | 12 (20.3%) |
| Respond to a relative's worries | 16 (33.3%) | 14 (23.7%) |

**Table S3.** Details of the changes in social or professional status and in family relationships for subjects who experienced change.

| Variable | Answer | Prospective (*N* = 92) | Retrospective (*N* = 81) |
|---|---|---|---|
| | | Frequency (%) | Frequency (%) |
| **Did the genetic test result** | | | |
| Change your professional plans? | No | 85/89 (95.5%) | 69/81 (85.2%) |
| | Yes | 2/89 (2.2%) | 12/81 (14.8%) |
| | Do not know | 2/89 (2.2%) | Not available |
| Complicate an application for a bank loan? | No | 78/87 (89.7%) | 66/81 (86.8%) |
| | Yes | 2/87 (2.3%) | 10/81 (13.2%) |
| | Do not know | 7/87 (8.0%) | Not available |
| Modify your sporting activities? | No | 58/90 (64.4%) | 28/81 (34.6%) |
| | Yes | 28/90 (31.1%) | 53/81 (65.4%) |
| | Do not know | 4/90 (4.4%) | Not available |
| **Did the genetic test result change your relationship with** | | | |
| Your partner? | No | 28/68 (41.2%) | 18/34 (52.9%) |
| | Yes | 40/68 (58.8%) | 16/34 (47.1%) |
| Your children? | No | 21/48 (43.8%) | 18/34 (52.9%) |
| | Yes | 27/48 (56.3%) | 16/34 (47.1%) |
| Your relatives with heart disease? | No | 18/67 (26.9%) | 7/34 (20.6%) |
| | Yes | 49/67 (73.1%) | 27/34 (79.4%) |
| Your relatives with the mutation but not yet ill? | No | 26/67 (38.8%) | 19/35 (54.3%) |
| | Yes | 41/67 (61.2%) | 16/35 (45.7%) |
| Your relatives without the mutation? | No | 30/68 (44.1%) | 19/35 (54.3%) |
| | Yes | 38/68 (55.9%) | 16/35 (45.7%) |
| Your relatives who do not know their genetic status? | No | 34/67 (50.7%) | 20/34 (58.8%) |
| | Yes | 33/67 (49.3%) | 14/34 (41.2%) |

**<u>Table S4.</u>** Descriptive analysis of STAI and distress (IES) in HCM patients versus other diseases.

| | STAI | | IES | |
|---|---|---|---|---|
| **Questionnaire** | **HCM** | **Other** | **HCM** | **Other** |
| **$Q_P$1** | 30.7 ± 9.9 | 30.5 ± 9.2 | 6.8 ± 10.4 | 7.0 ± 9.1 |
| **$Q_P$2** | 35.1 ± 12.6 | 34.4 ± 11.6 | 8.4 ± 10.7 | 9.1 ± 10.3 |
| **$Q_P$3** | 31.1 ± 11.5 | 28.9 ± 9.0 | 6.8 ± 10.9 | 6.2 ± 8.9 |
| **$Q_R$** | 35.8 ± 11.9 | 34.7 ± 11.6 | 10.4 ± 12.8 | 15.2 ± 14.8 |

Legend: HCM: hypertrophic cardiomyopathy.

**<u>Table S5.</u>** Direct comparisons between mutation carriers and non-carriers.

**(A) STAI state mean scores.**

| | STAI State Score | | |
|---|---|---|---|
| **Questionnaire** | **Non-Carriers** | **Mutation Carriers** | ***p*-Value (Student's *t*-test)** |
| **$Q_P$1** | 30.5 ± 9.3 | 30.6 ± 10.1 | 0.900 |
| **$Q_P$2** | 34.9 ± 12.7 | 34.4 ± 11.2 | 0.732 |
| **$Q_P$3** | 28.9 ± 9.9 | 31.7 ± 11.0 | 0.036 |
| **$Q_R$** | 34.8 ± 11.8 | 35.7 ± 11.7 | 0.529 |

**(B) Anxiety (STAI State score >35).**

| | Anxiety (STAI State Score >35) | | |
|---|---|---|---|
| **Questionnaire** | **Non-Carriers** | **Mutation Carriers** | ***p*-Value (Chi-Square Test)** |
| **$Q_P$1** | 41 (28.5 %) | 27 (28.7 %) | 0.967 |
| **$Q_P$2** | 55 (40.1 %) | 35 (38.5 %) | 0.799 |
| **$Q_P$3** | 29 (19.3 %) | 29 (29.3 %) | 0.069 |
| **$Q_R$** | 43 (36.8 %) | 49 (44.1 %) | 0.256 |

**(C) IES mean scores.**

| | IES Score | | |
|---|---|---|---|
| **Questionnaire** | **Non-Carriers** | **Mutation Carriers** | ***p*-Value (Student's *t*-test)** |
| **$Q_P$1** | 6.8 ± 10.7 | 7.0 ± 8.1 | 0.847 |
| **$Q_P$2** | 9.1 ± 11.1 | 8.0 ± 9.5 | 0.456 |
| **$Q_P$3** | 5.8 ± 9.7 | 7.6 ± 10.4 | 0.152 |
| **$Q_R$** | 10.0 ± 12.4 | 15.6 ± 15.0 | 0.003 |

**Table S6.** Summary of main studies of the psychosocial impact of predictive genetic testing in hereditary heart diseases (restricted to cardiomyopathies and arrhythmias). PGT: predictive genetic testing.

| **Study** | **Population** | **Mixed Phenotype** ***(Cardiac Disease Expressed or Not Expressed)*** **or Focused (*Asymptomatic Relatives Without Cardiac Phenotype)*** | **Longitudinal Design (Pre- and Post-PGT Evaluation) or Only Post-PGT** | **Study of Predictors of Anxiety or Psychological Distress** | **Period Between Results Disclosure and Post-PGT Psychosocial Evaluation** | **Main Finding** |
|---|---|---|---|---|---|---|
| Hendriks et al., 2008 | N= 77 | Mixed population | Yes, longitudinal | No | <18 months | Predictive testing for long QT syndrome consisting of cardiologic testing followed by molecular testing leads to distress, especially in carriers with an uncertain electrocardiogram at first visit. These distress levels return to normal at long term. However, for carriers with an uncertain electrocardiogram, the incidence of clinically relevant distress was high, most probably also caused by the consequences of having the disease. |
| Christiaans et al., 2009 | N= 228 | Mixed population | No, only post-PGT | Yes | Mean: 3.3 years | Quality of life and distress were worst in mutation carriers with manifest HCM before DNA testing and best in predictively tested mutation carriers without HCM. Illness and risk perception related variables were major determinants of QoL and distress. |
| Ingles et al., 2012 | N= 54 | Mixed population | Yes, longitudinal | No | 12 months | No change in health-related quality of life was observed up to 12 months after the result was given in patients and their asymptomatic family members |

| | | | | | | |
|---|---|---|---|---|---|---|
| | | | | | | undergoing genetic testing for an inherited heart disease. |
| Hickey et al., 2014 | N= 58 | Mixed population *(in fact, only with expressed cardiac disease)* | No, only post-PGT | No *(except cardiac symptom or expression)* | 18 months | Positive genetic results did not negatively impact patient well-being with the exception of the bodily pain domain of the SF-36. |
| Wynn et al., 2018 | N= 90 | Mixed population | No, only post-PGT | No | Mean: 17 months | Patients with positive genetic test results had higher scores for intrusive thoughts, avoidance, and distress when compared to those with negative genetic test results and were also more likely to make or plan to make life changes. 79% of participants reported complete satisfaction with the decision to have genetic testing. |
| Bordet et al., present study 2020 | N= 517 | Focused population (*only asymptomatic relatives without cardiac phenotype)* | Yes, longitudinal | Yes | Mean: 4.3 years | Medical benefit was not the main motivation, which emphasises the role of pre/post-test counselling. Only modest negative impacts of PGT were observed when performed by expert teams, but careful management is required in specific categories of subjects (especially history of depression or with high baseline anxiety), whatever the genetic test result. Few regrets about PGT were expressed. |

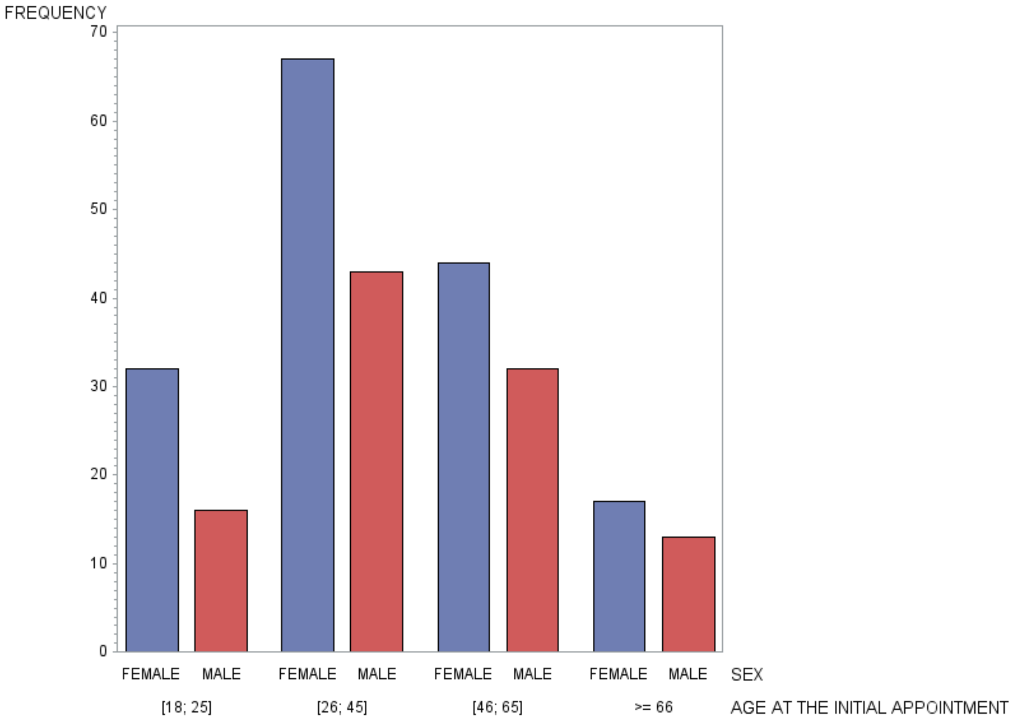


**(A)** Prospective cohort

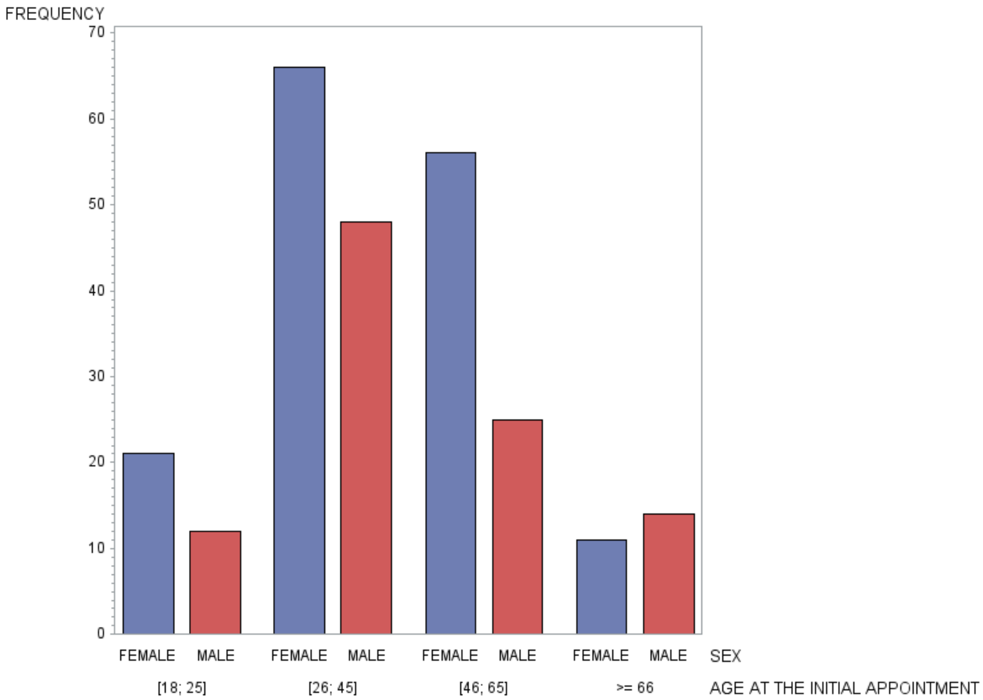


**(B)** Retrospective cohort

**Figure S1.** Distribution of age of subjects according to the sex in the prospective cohort (**A**) and retrospective cohort (**B**).

**A.** Prospective cohort

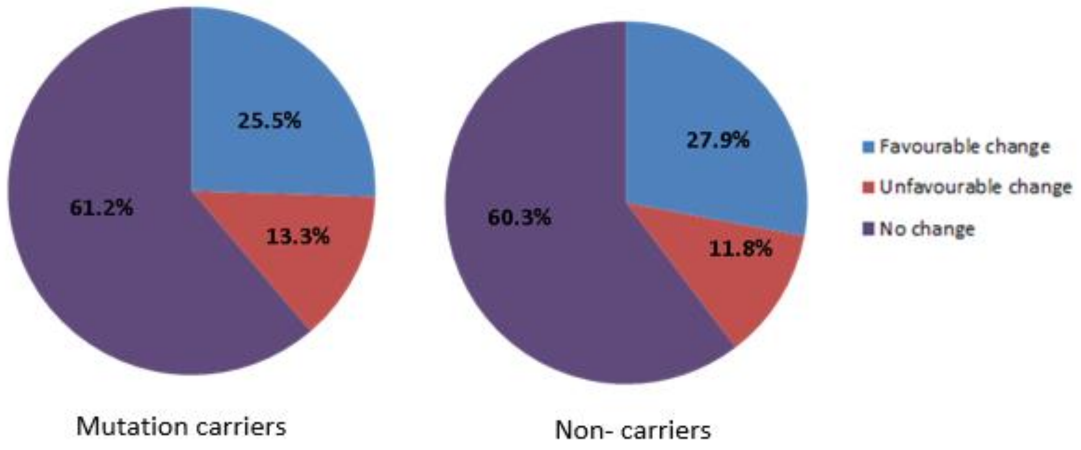


**B.** Retrospective cohort

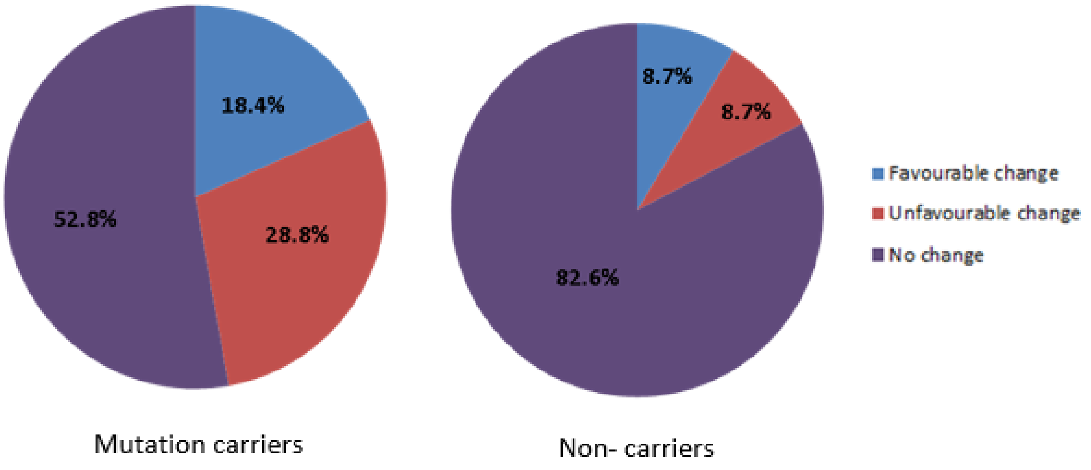


**Figure S2.** Social or professional changes and/or changes in family relationships in the prospective cohort (**A**) and retrospective cohort (**B**) for mutation carriers and non-carriers.

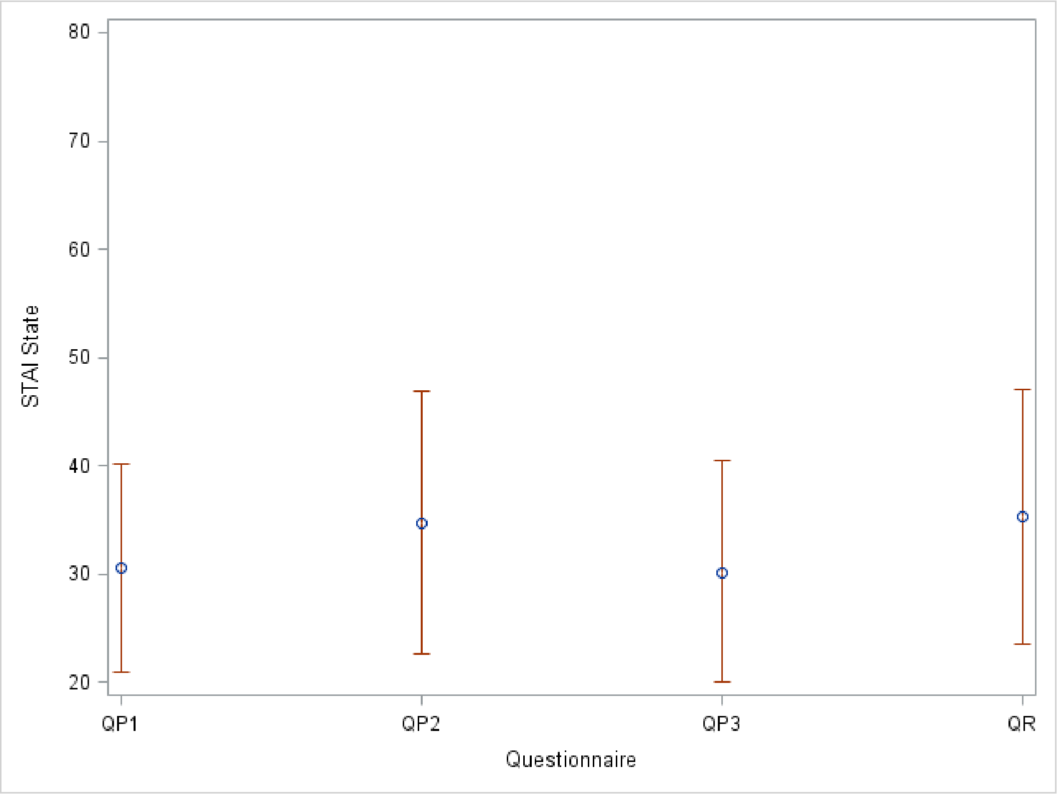


**<u>Figure S3.</u>** Change in the State-Trait Anxiety Inventory state score at Qp1, Qp2 and Qp3, and the STAI state score at Qr.

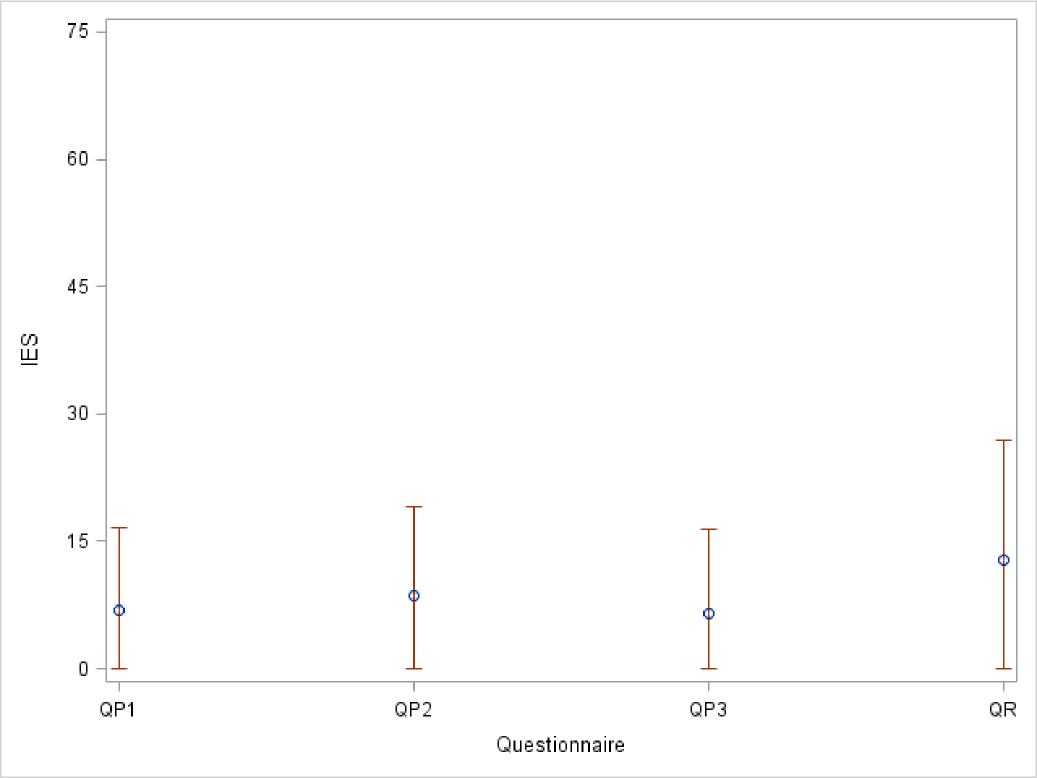


**<u>Figure S4.</u>** Change in the Impact of Event Scale score at Qp1, Qp2 and Qp3, and the IES score at Qr.
